# Therapeutic Effect of the Substrate-Selective COX-2 Inhibitor IMMA in the Animal Model of Chronic Constriction Injury

**DOI:** 10.3389/fphar.2018.01481

**Published:** 2018-12-18

**Authors:** Melissa Jones, Jie Wen, Prabhuanand Selvaraj, Mikiei Tanaka, Sean Moran, Yumin Zhang

**Affiliations:** ^1^Department of Anatomy, Physiology and Genetics, Uniformed Services University of the Health Sciences, Bethesda, MD, United States; ^2^Biomedical Instrumentation Center, Uniformed Services University of the Health Sciences, Bethesda, MD, United States

**Keywords:** substrate-selective COX-2 inhibitor, endocannabinoids, CB2 receptors, PGE_2_, chronic constriction injury, neuropathic pain

## Abstract

Enhancement of endocannabinoid signaling has emerged as an attractive strategy for the treatment of pain. In addition to the well-characterized hydrolytic pathways, cyclooxygenase-2 (COX-2) mediated oxygenation is thought to be an alternative route for endocannabinoid metabolism and therefore provides a new avenue for drug intervention. In this study, we examined the therapeutic effect of indomethacin morpholinamide (IMMA), a novel substrate-selective COX-2 inhibitor, in the chronic constriction injury (CCI) mouse model. Treatment with IMMA significantly alleviated hyperalgesia and mechanical allodynia demonstrated by increased thermal withdrawal latency in Hargreaves test and tactile thresholds in Von Frey test. Accumulation of astrocytes and microglia in spinal cord dorsal horn and infiltration of macrophages into the dorsal root ganglion and sciatic nerve were reduced by drug treatment. Co-administration of the CB2 receptor antagonist, but not the CB1 receptor antagonist partially reversed the inhibitory effect of IMMA on pain sensitivity and inflammatory infiltrates. IMMA downregulated the mRNA expression of TNF-α and IL-1β and the production of IL-6 and MCP-1 proteins in the ipsilateral sciatic nerve. The enhanced NF-κB DNA binding activity in the CCI mouse dorsal spinal cord was also significantly reduced, suggesting that inactivation of NF-κB contributes to the anti-inflammatory property of IMMA. However, different from the previous reports showing that IMMA can increase endocannabinoids without interfering with arachidonic acid metabolism, treatment with IMMA failed to elevate the endogenous levels of AEA and 2-AG, but significantly reduced the production of prostaglandin E_2_ (PGE_2_). Furthermore, the mRNA expression of enzymes involved in PGE_2_ production, COX-2 and prostaglandin E synthase 2 in the ipsilateral sciatic nerve was also suppressed by IMMA treatment. Taken together, these results suggested that IMMA might exert anti-nociceptive effects through multiple mechanisms which include, but are not limited to, CB2 receptor activation and reduced PGE_2_ production.

## Introduction

Neuropathic pain is a debilitating form of chronic pain caused by traumatic nerve injury, toxic insults and various disease states (Donvito et al., 2018). The pathogenesis of neuropathic pain is complex, and is likely caused by the aberrant synaptic neurotransmission, enhanced inflammatory response, and neuronal injury. Neuropathic pain is unmanageable with the current available drugs such as opioids and non-steroidal anti-inflammatory drugs (NSAID), therefore development of novel therapeutic approach is an area of greater unmet clinical need (Marchand et al., 2005).

Enhancement of endocannabinoid signaling has recently emerged as an attractive strategy for the treatment of pain (Guindon and Hohmann, 2009; Kinsey et al., 2009, 2010; Schlosburg et al., 2009; Maldonado et al., 2016; Donvito et al., 2018). The endocannabinoid system is composed of CB1 and CB2 cannabinoid receptors, their endogenous ligands anandamide (AEA) and 2-arachidonyl glycerol (2-AG), and enzymes for their synthesis and degradation (Di Marzo, 2009; Stella, 2009). Unlike the other neurotransmitters, endogenous cannabinoids are not stored in vesicles and are produced when/where they are needed. This “on demand” synthesis suggests that the production of endocannabinoids is able to activate cannabinoid receptors in a time- and region-specific manner without activating all accessible receptors indiscriminately (Hwang et al., 2010). Following the induction of inflammatory and neuropathic pain, cannabinoid receptors and their endogenous ligands AEA and 2-AG are increased in dorsal root ganglion (DRG) and spinal cord dorsal horn on the ipsilateral side of injury (Mitrirattanakul et al., 2006; Drew et al., 2009). AEA is also found in the inflamed paw following carrageenan-induced inflammation (Holt et al., 2005). In the formalin model, selective inhibitor of 2-AG hydrolysis increases the level of 2-AG to exert analgesic effect (Bisogno et al., 2009). All of these findings indicate that inflammatory and neuropathic pain involves peripheral and central endocannabinoid alteration, suggesting that manipulation of the endocannabinoid system may play a predominant role in the pain treatment.

In addition to inactivation by monoacylglycerol lipase (MAGL) and fatty acid amide hydrolase (FAAH), 2-AG and AEA can also be oxygenated by cyclooxygenase 2 (COX-2) to generate prostaglandin glycerol esters (PG-Gs) and prostaglandin ethanolamides (PG-EAs) (Rouzer and Marnett, 2011). The production of PG-G and PG-EA causes inflammation, excitotoxicity, and results in inflammatory and neuropathic pain (Sang et al., 2007; Gatta et al., 2012). It has been reported that COX-2 expression is upregulated in chronic pain states (Hay and de Belleroche, 1997; Samad et al., 2001; Guay et al., 2004), implying that under these conditions, endocannabinoid oxygenation by COX-2 might be enhanced. Therefore, it is conceivable that the therapeutic efficacy of endocannabinoids can be counteracted by the metabolites of endocannabinoid oxygenation. Recent studies have demonstrated that many weak competitive inhibitors of arachidonic acid (AA) metabolism are potent inhibitors of endocannabinoid oxygenation. R-enantiomers of ibuprofen, naproxen and flurbiprofen, which are considered inactive as COX-2 inhibitors, are potent ’substrate-selective COX-2 inhibitors’ (SSCIs) of endocannabinoid oxygenation (Duggan et al., 2011). SSCIs inhibit COX-2 activity when AEA and 2-AG, but not AA, are used as substrates. Therefore, the use of SSCIs is likely to avoid the side effects caused by the traditional NSAID, which include cardiovascular risk and gastrointestinal bleeding (Duggan et al., 2011; Hermanson et al., 2014).

A recent study found that LM-4131 (which is also named IMMA), the morpholinamide of indomethacin, is a novel SSCI that reduces anxiety-like behaviors by selectively increasing the endogenous levels of AEA without affecting the levels of non-endocannabinoid lipids, such as oleoylethanolamide and palmitoylethanolamide, and the prostaglandin synthesis (Hermanson et al., 2013). Given the demonstrated efficacy of the inhibitors of endocannabinoid hydrolysis in the management of inflammatory and neuropathic pain, we speculated that inhibition of 2-AG and AEA oxygenation by IMMA could provide a novel strategy for pain treatment. In this study, we employed the animal model of chronic constriction injury to investigate how IMMA impacts the hyperalgesia and allodynia behavior and the underlying mechanisms.

## Materials and Methods

### Materials

Indomethacin morpholinamide, AM281, a CB1 receptor antagonist and AM630, a CB2 receptor antagonist and the deuterated AEA (AEA-d4), 2-AG (2-AG-d5), and AA (AA-d8) were purchased from Cayman Chemicals (Ann Arbor, MI, United States). All other chemicals and reagents were purchased from Sigma (St. Louis, MO, United States), unless stated otherwise.

### Animals

Male, 8–10 weeks old C57BL/6J mice were purchased from the Jackson Laboratory (Bar Harbor, ME, United States). Animal care and experimental procedures were carried out in accordance with NIH guidelines and approved by the Uniformed Services University Animal Care and Use Committee.

### Chronic Constriction Injury Surgery

The surgical procedure for partial sciatic nerve ligation was carried out as described previously (Bennett and Xie, 1988) with slight modifications (Wen et al., 2018). Mice were anesthetized with isoflurane (3.5% for induction and 2.0% for maintenance) and the middle to lower back above the dorsal left thigh were shaved and cleaned with iodine and 75% ethanol in preparation for surgery. Using these aseptic procedures, the common sciatic nerve was exposed at the mid-thigh level by blunt dissection. Under a dissection microscope, a nerve segment of 5 mm long was separated from the surrounding tissue. Two ligatures of 6-0 sterile silk, spaced 1.0 to 1.5 mm apart, were loosely tied around the sciatic nerve. In sham-operated mice, the sciatic nerve was isolated and exposed without ligation. The muscles and the skin were closed with sutures.

### Assessment of Nociceptive Behavior

#### Hargreaves Test

The thermal escape latency was determined using the Hargreaves type thermal escape testing system (Plantar Analgesia Meter, IITC Life Science Inc.) as we previously described (Wen et al., 2018). Briefly, mice were placed individually in Plexiglas cubicles on a glass surface and allowed to acclimate for 30 min. The light beam was focused on the bottom of the glass and created an intense spot under the foot pad with the aid of an angled mirror. Paw withdrawal latency was defined as the time required for the paw to show an abrupt withdrawal. In the absence of a response at 20 s, the stimulus was terminated, and that latency was assigned.

#### Von Frey Test

Mechanical allodynia induced by partial ligation of the sciatic nerve was assessed with the “Von Frey” test as described (Smith et al., 2006). The level of allodynia was determined by testing the withdrawal response to tactile stimuli with Von Frey filaments of varying thickness. Mechanical thresholds were determined by the “Up-Down” methods (Chaplan et al., 1994). Mice were placed in a Plexiglas cage with mesh metal flooring and allowed to acclimate for 30 min before testing. A series of calibrated Von Frey filaments (Stoelting, Wood Dale, IL, United States) with logarithmically incremental stiffness ranging from 2.44 to 4.31 were applied to the mid plantar surface of the hind paws. Each hair was presented perpendicularly against the paw, with sufficient force to cause slight bending, and held for 3 s. A positive response was noted if the paw was sharply withdrawn. Flinching immediately upon removal of the hair was also considered a positive response. In case of ambiguous response such as ambulation, the stimulus was repeated.

### Drug Treatment

All drugs were dissolved in DMSO-cremophor-saline (1:1:18), which was used as a vehicle control. CCI and sham control mice were randomly assigned to receive IMMA or vehicle. Drugs were given intraperitoneally (i.p.) 3 h after surgery and then once a day for 7 or 14 days. Animals were sacrificed on day 7 or day 14 and the sciatic nerve, spinal cord and DRG were collected for further analysis. To determine the cannabinoid receptor dependency, IMMA (10 mg/kg) was co-administered with the CB1R antagonist AM281 (3 mg/kg, i.p.) and the CB2R antagonist AM630 (3 mg/kg, i.p.), respectively.

### qRT-PCR

On day 7 after CCI surgery, mice were euthanized and the sciatic nerves were dissected out. Total RNA was extracted from tissues by using Tri reagent. RNA (1 μg) was reverse transcribed to cDNA and then subjected to PCR. The reaction mixture contained TaqMan fast universal PCR master mix (Applied Biosystems, Grand Island, NY, United States), 50 ng cDNA and 100 nM of each primer. qRT-PCR was run in a CFX96^TM^ Real Time System (Bio-Rad, Hercules, CA, United States). The cycling condition was 95°C for 15 s, 75°C for 1 min, 55°C for 30 s for 40 cycles, followed by a melting point determination program. The relative expression level of each gene was determined by the 2^-ΔCt^ method and normalized by GAPDH. The following primers were used: CB2 (NM_001305278): forward, 5′-tgctgtcatatgctggtc-3′ and reverse, 5′-atcctggctcctaggtggtt-3′; DAGLβ (NM_144915.3): forward, 5′-actcagatttgcctgcctct-3′ and reverse, 5′-gcctacaagtcccaacacct-3′; IL-1β (BC011437): forward, 5′-gcaactgttcctgaactcaact-3′ and reverse, 5′-atcttttggggtccgtcaact-3′; TNF-α (NM_013693): forward, 5′-ccctcacactagatcatcttct-3′ and reverse, 5′-gctacgacgtgggctacag-3′; MCP-1 (BC145869): forward, 5′-cagcaagatgtcccaatga-3′ and reverse, 5′-tctggacccattccttcttg-3′; COX-2 (BC052900): forward, 5′-gtggaaaaacctcgtccaga-3′ and reverse, 5′-gctcggcttccagtattgag-3′; PGES2 (NM133783): forward, 5′- acttccactccctgccctat-3′ and reverse, 5′-gttgcaagctgtctccttcc-3′; EPR2 (BC005440): forward, 5′-atgctcctgctgcttatcgt-3′ and reverse, 5′-agggcctcttaggctactgc-3′; and GAPDH (GU214026): forward, 5′- aggtcggtgtgaacggatttg-3′ and reverse, 5′-tgtagaccatgtagttgaggtca-3′.

### Immunostaining

Animals were euthanized by an intraperitoneal injection of ketamine (90 mg/kg) and xylazine (10 mg/kg) at a volume of 10 μl/g solution and then intracardially perfused with ice cold 1× PBS followed by 4% paraformaldehyde in the same buffer. Sciatic nerve, DRG and spinal cord tissues were dissected out and kept in 4% paraformaldehyde at 4°C overnight. Tissues were then transferred into 1× PBS containing 30% sucrose at 4°C overnight and stored at -80°C after being embedded in Tissue-Tek O.C.T. compound (Sakura Finetek USA, Inc., CA, United States). Transverse sections of lumbar spinal cords were cut at 14 μm using a cryostat (Leica model CM1900, Bannockburn, IL, United States) and mounted onto Superfrost Plus slides for immunoassay. Primary antibodies including anti-rat Iba1 (1:300; Abcam, Cambridge, MA, United States), anti-mouse GFAP (1:500; Cell Signaling, Danvers, MA, United States), anti-mouse F4/80 (1:300; eBioscience, San Diego, CA, United States), and anti-rabbit NF-κB (1:200; Full Moon Biosystems, Inc., Sunnyvale, CA, United States) were used for immunofluorescence assay. Briefly, the slides were washed with 1× PBS twice, and then incubated in 1× PBS containing 5% donkey serum and 0.3% Triton X-100 for 30 min at room temperature, followed by respective primary antibody incubation in the same buffer overnight. The slides were then washed with 1× PBS containing 0.2% Triton X-100 and incubated with Alexa Fluor 488 or Alexa Fluor 594 conjugated donkey anti-rabbit, mouse or rat secondary antibodies (1:750; Thermo Fisher Scientific, Waltham, MA, United States) for 1 h. The slides were again washed with 1× PBS twice and covered with Fluoroshield mounting medium with DAPI. Immunofluorescence images shown in Figures 2, 3, and 5 were obtained with a fluorescence microscope (Nikon Eclipse TE-2000 U). The cells with both DAPI and expected fluorescence were defined as positively stained cells. All immunofluorescence data were obtained in a minimum of 5–7 serial sections from the lumbar spinal cord, DRG and sciatic nerve of each animal. The positive stained cells in the region of interest (ROI) were included. Immunoreactivity positive cells of F4/80 were counted and expressed as mean cell numbers per mm^2^. The fluorescence intensity of GFAP, Iba1, and p-NF-κB was quantified with NIH Image J software. Negative controls were routinely performed in which the primary antibodies were omitted.

### ELISA Assay

For assay of IL-6 and MCP-1, the whole cell lysates from fresh ipsilateral sciatic nerves of CCI mice 7 days after surgery were prepared with RIPA buffer (10 mM Tris–HCl pH 7.4, 30 mM NaCl, 1 mM EDTA, 1% Nonidet P-40, supplemented with 1 mM Na_3_VO_4_, 1 μg/ml leupeptin, 1 μg/ml pepstatin A, 1 μg/ml aprotinin and 1 mM PMSF). Each sample was employed in duplicate to quantify IL-6 and MCP-1 according to the manufacturer’s protocol (Thermo Fisher Scientific, Waltham, MA, United States). For the measurement of NF-κB activity, nuclear extracts were obtained with NE-PER nuclear extraction kit (Thermo Fisher Scientific, Waltham, MA, United States). Approximately 10 mg of spinal cord tissue of CCI mice 7 days after surgery were homogenized in the CER I buffer provided in the kit and incubated on ice for 10 min. CER II buffer was then added to the tubes containing homogenized tissue and centrifuged for 5 min at maximal speed. After removing the supernatant, the pellets were resuspended in ice-cold nuclear extraction reagent and vortexed on ice. Then the tube was centrifuged at maximal speed for 10 min and supernatant containing the nuclear extract was applied for NF-κB activity assay (Abcam, ab133112, Cambridge, MA, United States). For PGE_2_ assay, mouse sciatic nerve was homogenized with 40 μl of 0.02% trifluoroacetic acid (TFA) and 100 μl of acetonitrile on ice and dispersed in 1 ml of acetonitrile by vortex and left at 4°C overnight. On the second day the homogenate-acetonitrile mixture was centrifuged at 2,000 *g* for 5 min to remove the debris and the supernatant was transferred to a silanized glass tube. The collected supernatant was subject to evaporation under the nitrogen gas streaming in a water bath (approx. 35°C), then reconstituted with acetonitrile. PGE_2_ production in the lipid extract was measured with PGE_2_ EIA kit following the manufacturer’s protocol (Cayman Chemical, Ann Arbor, MI, United States).

### LC-MS/MS Analysis for 2-AG, AEA, and AA

The sciatic nerve tissue obtained from mice 7 days post-CCI was homogenized with 40 μl of 0.02% TFA, 250 μl of acetonitrile, and 25 picomoles of 2-AG-d5 and AA-d8 and 0.5 picomoles of AEA-d4 (Cayman Chemical) using a Potter homogenizer under 4°C environment. The homogenate was dispersed completely in 2.5 ml acetonitrile by vortex and kept at 4°C overnight. The homogenate was subjected to centrifugation at 2,000 *g* × 5 min to remove the debris and then the supernatant was evaporated under the nitrogen gas streaming in a water bath (approx. 35°C). The lipid was re-suspended with 100 μl of acetonitrile and stored at -80°C until use.

An HPLC system (1200 Series, Agilent Technologies, Santa Clara, CA, United States) was used with a reverse phase guard column (Wide Pore C18 (ODS), 4 mm × 2 mm ID; Phenomenex, Torrance, CA, United States) and the reverse phase column (Sephasil Peptide C18, 5 μ, ST, 100 mm × 4.6 mm ID; Pharmacia Biotech, Piscataway, NJ, United States) was maintained at 40°C. The mobile phase was composed of solvent A (0.2% formic acid in water) and solvent B (0.2% formic acid in methanol), and the following gradient was used: 62% A/38% B isocratic for 30 s, ramp to 90% B in 60 s, isocratic at 90% B for 18.5 min., ramp back to 62% A/38% B in 60 s, and re-equilibrate at 62% A/38% B for 8 min. The flow rate was 0.4 ml/min. The HPLC output was directed into the TurboV electrospray ionization (ESI) source of a Q-Trap 4000 mass spectrometer (AB Sciex, Framingham, MA, United States). The injection volume was 20 μl. LC-MS/MS analysis was performed in a positive mode with the ion source temperature of 600°C, a spray voltage of 5.5 kV and a declustering potential of 45 V. Multiple reactions monitoring (MRM) was performed on the transitions *m*/*z* 379 → 287 for 2-AG, 384 → 292 for 2-AG-d5, 348 → 62 for AEA, 352 → 66 for AEA-d4, 305 → 93 for AA and 313 → 229 for AA-d8. The concentrations of 2-AG, AEA, and AA were determined by calculating the corresponding peak area ratio to the internal standard (IS) using a linear fit weighting to the calibration curve.

### Statistical Analysis

Data were analyzed for statistical significance by using one-way analysis of variance (ANOVA) or two-way ANOVA. Tukey-Kramer *post hoc* analysis was used for comparing different treatment groups. Results were presented as mean ± standard error of the mean (SEM). A significant difference was determined as *p* < 0.05.

## Results

### IMMA Dose Dependently Attenuated Neuropathic Pain in the CCI Mouse Model

**FIGURE 1:**
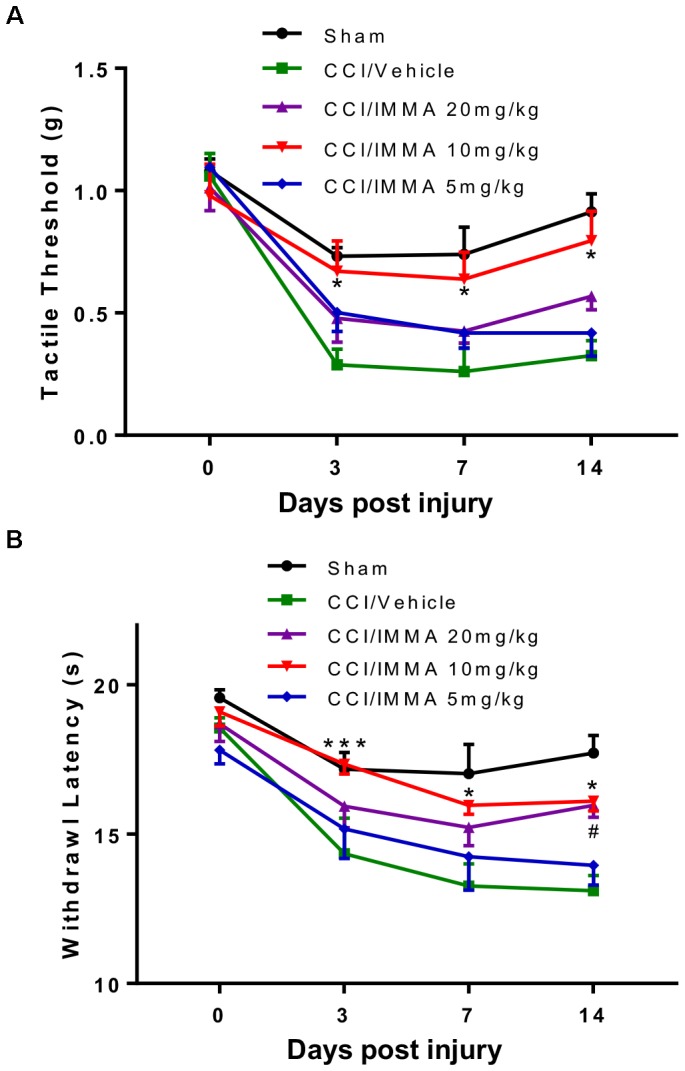
IMMA dose dependently alleviated neuropathic pain in CCI mice. At days 0, 3, 7, and 14 after surgery, mechanical tactile thresholds were evaluated by the “Up-Down” method using Von Frey filaments **(A)** and thermal withdrawal latency was determined by Hargreaves test **(B)**. Treatment with IMMA at 10 mg/kg significantly reduced thermal withdrawal latency and increased mechanical thresholds compared to the vehicle group (^∗^*p* < 0.05 and ^∗∗∗^*p* < 0.001. *n* = 15/group). At 5 mg/kg, IMMA had no effect, although there was a trend toward the increased tactile threshold and withdrawal latency. Treatment of IMMA at 20 mg/kg significantly increased thermal withdrawal latency only on day 14 compared to the vehicle group (^#^*p* < 0.05. *n* = 15/group). Data were analyzed using two-way ANOVA, and the Tukey-Kramer *post hoc* test was used for comparing different treatment groups.

To investigate the therapeutic effect of IMMA in neuropathic pain, different doses (5, 10, and 20 mg/kg) of IMMA were administrated *i.p.* to the CCI operated mice 3 h after injury and then once a day until the mice were sacrificed. Hargreaves and Von Frey tests were performed at 3, 7, and 14 days post-CCI (Figure 1). Treatment with IMMA at 10 mg/kg significantly attenuated neuropathic pain at all time points. 10 mg/kg IMMA application greatly increased mechanical tactile thresholds with values of 0.67, 0.64, and 0.8 g compared to the CCI vehicle with values of 0.28, 0.26, and 0.22g at days 3, 7, and 14 post CCI surgery, respectively (Figure 1A; ^∗^*p* < 0.05). Thermal withdrawal latency was also significantly augmented in the treatment group with the values of 17.4, 15.6, and 16.1 s compared to the CCI group with the values of 14.4, 13.3, and 13.1 s at days 3, 7, and 14 post CCI (Figure 1B; ^∗∗∗^*p* < 0.001; ^∗^*p* < 0.05). For the dose of 20 mg/kg, a significant increase of thermal withdrawal latency was only observed on day 14 in the IMMA treatment group with the value of 15.6 s compared to the CCI vehicle group with the value of 13.1 s (Figure 1B; ^#^*p* < 0.05). There were no significant differences in both thermal hyperalgesia and mechanical allodynia between the 5 mg/kg treatment group and the CCI vehicle group (Figures 1A,B). Therefore, the 10 mg/kg of IMMA seemed to be optimal and was used for the rest of experiments.

### Therapeutic Effect of IMMA on Neuropathic Pain Is Partially Mediated by CB2 Cannabinoid Receptor

**FIGURE 2:**
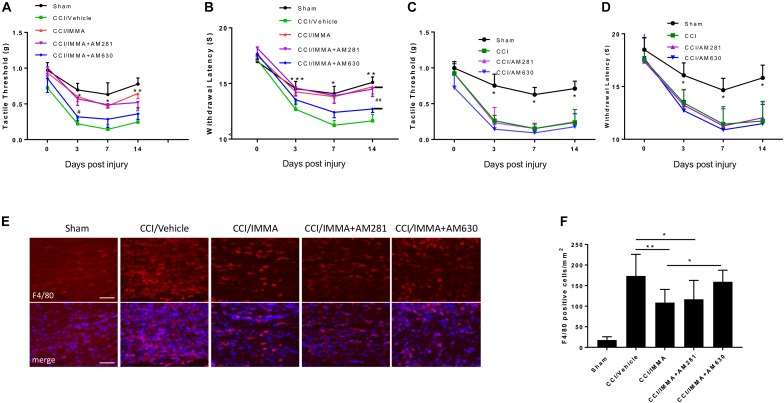
The analgesic effects of IMMA in CCI mice were partially mediated by CB2 cannabinoid receptor. Co-administration of IMMA with the CB1 receptor antagonist AM281 in the CCI mouse resulted in similar anti-allodynic and anti-hyperalgesia effects to the IMMA alone treatment group **(A,B)**. Treatment with IMMA and the CB2 receptor antagonist AM630 exhibited a tendency of reduced mechanical thresholds compared to the IMMA alone group (**A**; ^∗^*p* < 0.05, ^∗∗^*p* < 0.01 compared between CCI/vehicle and CCI/IMMA; ^#^*p* < 0.05 between CCI/IMMA and CCI/IMMA plus AM630, *n* = 10/group). The thermal withdrawal latency was significantly reduced in IMMA and AM630 treatment group compared to the IMMA alone group (**B**; ^∗^*p* < 0.05; ^∗∗^*p* < 0.01; and ^∗∗∗^*p* < 0.001 were obtained when the CCI/vehicle group was compared to the IMMA treatment groups; ^##^*p* < 0.01 compared between IMMA group and IMMA plus AM630 treatment group, *n* = 10/group). Cannabinoid receptor antagonists alone had no effect on neuropathic pain. The CCI mice treated with AM281 or AM630 exhibited similar tactile threshold **(C)** and thermal withdrawal latency **(D)** to the CCI group. ^∗^*p* < 0.05 was obtained when the CCI/vehicle, AM281 and AM630 treated groups were compared to the sham group (*n* = 7/group). Macrophage infiltration to the injured sciatic nerve at 14 days post-CCI was also significantly reduced by IMMA treatment, and this effect was reversed by addition of AM630, but not AM281 (**E,F**; ^∗^*p* < 0.05; ^∗∗^*p* < 0.01; and ^∗∗∗^*p* < 0.001. *n* = 6/group). Data obtained for neuropathic pain behaviors **(A–D)** were analyzed using two-way ANOVA, and one-way ANOVA was used for quantifying the differences in F4/80 staining among the various groups **(F)**. The Tukey-Kramer *post hoc* test was used for all the comparison. The merged images in **E** showed the co-localization of F4/80 (red) and DAPI (blue for nuclei staining). Scale bar = 250 μm.

In order to evaluate the involvement of endocannabinoid signaling in IMMA treatment for neuropathic pain, a combination of IMMA together with the CB1R antagonist AM281 or the CB2R antagonist AM630 was administered to the CCI mice 3 h after injury and then once a day until day 14. Similar to the previous results (Figure 1), IMMA significantly increased mechanical tactile thresholds (^∗^*p* < 0.05; ^∗∗^*p* < 0.01; Figure 2A) and thermal withdrawal latency (^∗^*p* < 0.05; ^∗∗^*p* < 0.01; ^∗∗∗^*p* < 0.001; Figure 2B). Here, we found that CCI mice treated with IMMA and AM281 showed similar hyperalgesia behavior as seen in the IMMA treatment group. Compared to the tactile thresholds of 0.22 and 0.25 g in the CCI vehicle group at days 3 and 14 post surgery, IMMA and AM281 treatment group exhibited an obviously reduced pain hypersensitivity with the Von Frey test values of 0.57 and 0.52, respectively (Figure 2A). Treatment with IMMA and AM281 also produced the same degree of anti-nociceptive effect as the IMMA alone in the Hargreaves test. At days 3 and 7 post CCI, treatment with IMMA and AM281 increased withdrawal latency compared to the CCI vehicle group, and on day 14 a significant increase of withdrawal latency was observed with the value of 14.48 s compared to the value of 11.63 in the vehicle group. These observations indicated that addition of CB1R antagonist didn’t affect the therapeutic efficacy of IMMA. On the other hand, the CB2R antagonist partially reversed the IMMA’s therapeutic effect on neuropathic pain; with regard to the mechanical stimulation, the addition of AM630 significantly counteracted IMMA mediated increase of tactile thresholds at days 3 and 14 after CCI (^#^*p* < 0.05; Figure 2A). AM630 addition also showed an opposite effect on thermal hyperalgesia thresholds compared to the IMMA alone at all time points, despite that a significant difference between the IMMA and IMMA plus AM630 treatment groups was only observed at 14 days post injury (^##^*p* < 0.01; Figure 2B). Consistent with our previous findings (Wen et al., 2018), the CCI group treated with either AM281 or AM630 displayed the similar mechanical allodynia and thermal hyperalgesia to the CCI/vehicle group (Figures 2C,D), indicating that the cannabinoid receptor antagonists alone do not have any effect to alleviate neuropathic pain.

To further determine the role of cannabinoid receptors in the therapeutic action of IMMA, we also examined macrophage infiltration in the ipsilateral sciatic nerve. As shown in Figures 2E,F, a significant increase of F4/80 positive cells was observed in the CCI vehicle group compared to the sham control group. IMMA treatment dramatically reduced F4/80 positive cells with 109 cells/mm^2^ compared to 173 cells/mm^2^ in the vehicle group (Figure 2F). In agreement with the findings from the behavior test, AM630, but not AM281, reversed the IMMA effect on blocking macrophage infiltration into the sciatic nerve with 150 positive cells/mm^2^ compared to 109 positive cells/mm^2^ in the IMMA treatment group (*p* < 0.05).

### IMMA Treatment Suppressed the Number of Inflammatory Cells in the Spinal Cord Dorsal Horn and Dorsal Root Ganglion of CCI Mice

**FIGURE 3:**
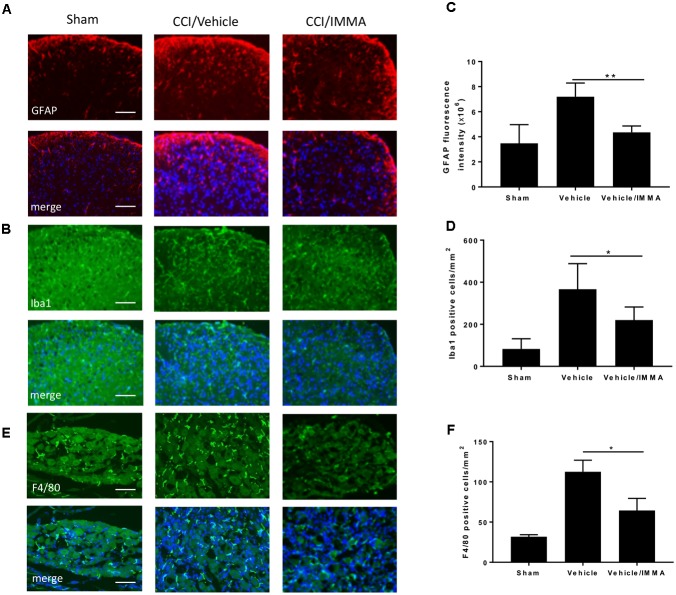
Accumulation of inflammatory cells in CCI mouse spinal cord dorsal horn and DRG was reduced by IMMA treatment. The numbers of GFAP and Iba1 positive cells in ipsilateral lumbar spinal cord dorsal horn **(A,B)** and F4/80 positive staining in ipsilateral DRG **(E)** in IMMA treated group were remarkably reduced compared to the CCI/vehicle group. Quantitation of the GFAP and Ibal positive cells in spinal cord dorsal horn and F4/80 positive cells in DRG were shown in **C,D,F** (^∗^*p* < 0.05 and ^∗∗^*p* < 0.01, *n* = 10/group). One-way ANOVA was used for quantifying the differences in GFAP, Iba1, and F4/80 staining among the sham, vehicle and IMMA treated groups **(C,D,F)**. The Tukey-Kramer *post hoc* test was used for all the comparison. The merged images in **A,B,E** showed the co-localization of DAPI (blue) with GFAP (red), Iba1 (green), and F4/80 (green). Scale bar = 250 μm.

In addition to examining macrophage infiltration in the injured sciatic nerve, we also investigated the effect of IMMA on other regions that are involved in pain processing. The lumber spinal cord and DRG from mice 14 days after CCI were fixed with paraformaldehyde and subjected to immunofluorescence staining. Compared to the sham control, the CCI/vehicle group displayed strong fluorescence signals in GFAP, Ibal and F4/80 staining, the respective markers of astrocytes, microglia, and microglia/macrophages. IMMA treatment remarkably suppressed the accumulation of those inflammatory cells (Figures 3A,B in spinal cord dorsal horn and Figure 3E in DRG). Quantification of the GFAP fluorescence intensity and the counting of microglia/macrophages in the spinal cord dorsal horn and DRG revealed a 2–3 folds increase of these inflammatory cells, and the increase was significantly reduced by IMMA treatment (Figures 3C,D,F).

### IMMA Alleviated Inflammatory Response in the Ipsilateral Sciatic Nerve

**FIGURE 4:**
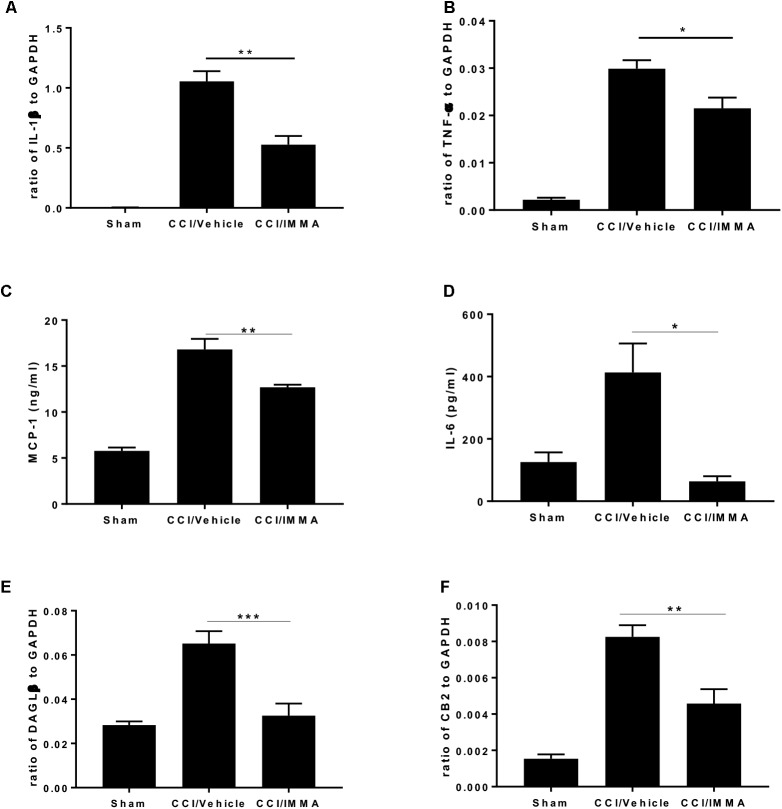
. IMMA treatment attenuated the production of proinflammatory cytokines and chemokines in the ipsilateral sciatic nerve of CCI mouse. The fresh sciatic nerves from mice 7 days post-surgery were subjected to qRT-PCR analysis and ELISA. The remarkably increased IL-1β **(A)**, TNF-α **(B)**, DAGLβ **(E)**, and CB2R **(F)** expression was observed in the CCI/vehicle group and reduced by IMMA treatment. Protein levels of MCP-1 **(C)** and IL-6 **(D)** in the ipsilateral sciatic nerve 7 days post-surgery were significantly elevated by CCI and inhibited by IMMA treatment (^∗^*p* < 0.05, ^∗∗^*p* < 0.01; and ^∗∗∗^*p* < 0.001. *n* = 10/group). One-way ANOVA was used for comparing the differences among the sham, vehicle and IMMA treated groups. The Tukey-Kramer *post hoc* test was used for the comparison.

Analysis of inflammatory cytokines IL-1β and TNF-α mRNA transcripts showed that sciatic nerve ligation induced a strong inflammatory response in the injured site compared to the sham. The mRNA levels of these genes were significantly increased in the CCI vehicle group and reduced in the IMMA treatment group (Figures 4A,B). Consistently, the increased production of MCP-1 and IL-6 in the injured sciatic nerve examined by ELISA was also significantly reduced by the drug treatment (Figures 4C,D).

In agreement with the notion that DAGLβ and CB2 receptor are primarily expressed in macrophages and microglia (Hsu et al., 2012; Shin et al., 2018), we found a significant increase of DAGLβ and CB2R expression in the ipsilateral sciatic nerve of the CCI vehicle group and the increased expression was significantly attenuated by IMMA treatment (Figures 4E,F).

### Inactivation of NF-kB Contributed to IMMA Mediated Anti-Inflammatory Function

**FIGURE 5:**
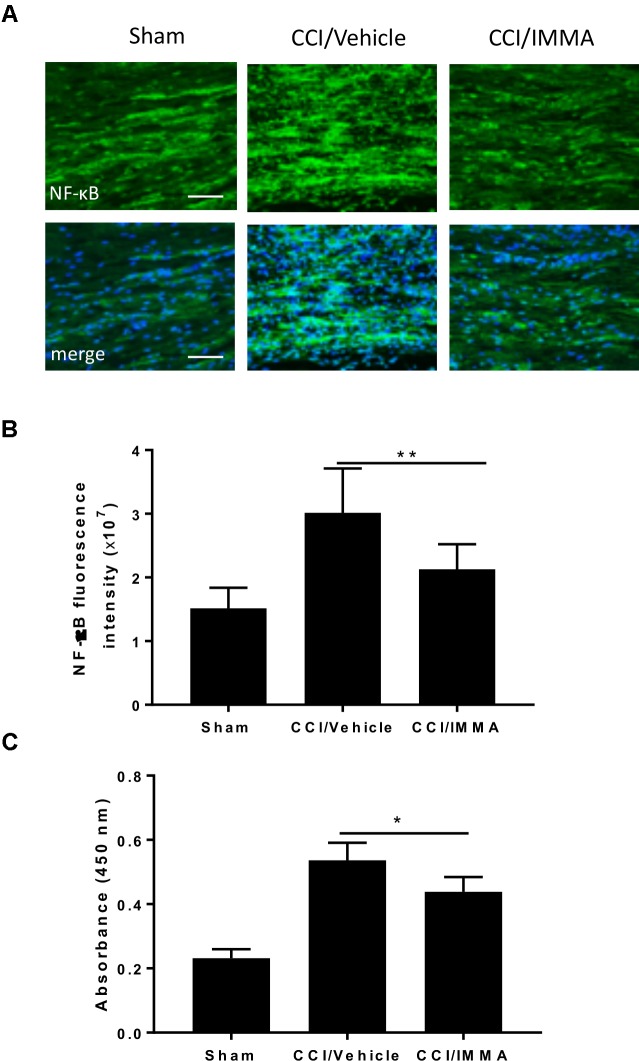
Treatment with IMMA attenuated the phosphorylation of NF-κB in ipsilateral sciatic nerve and the NF-κB DNA binding activity in dorsal spinal cord of CCI mice. Activation of NF-κB was detected by immunofluorescence staining with an antibody against phosphorylated NF-κB in the ipsilateral sciatic nerve of CCI mice 7 days after surgery. Positive phosphorylated NF-kB staining in sciatic nerve was significantly greater in the CCI/vehicle group than that in the sham group and dramatically reduced by IMMA treatment **(A)**. The fluorescence intensity of NF-kB positive staining cells showed a twofold increase in the CCI/vehicle and significantly reduced by IMMA **(B)**. NF-κB DNA binding activity was evaluated by ELISA using fresh spinal cord from mice 7 days after surgery. CCI/vehicle group showed an enhanced DNA binding activity compared to the sham, which was reduced by IMMA treatment **(C)**. ^∗^*p* < 0.05 and ^∗∗^*p* < 0.01 (*n* = 10/group). One-way ANOVA was used for quantifying the differences in NF-kB fluorescence intensity **(B)** and absorbance at 450 nm **(C)** among the sham, vehicle and IMMA treated groups. The Tukey-Kramer *post hoc* test was used for all the comparison. The merged images in **(A)** showed the co-localization of DAPI (blue) with NF-kB (green). Scale bar = 250 μm.

NF-κB is a major transcriptional factor responsible for the upregulation of inflammatory cytokines. Here, we employed immunofluorescence staining and ELISA assay to evaluate NF-κB activity in the IMMA treated CCI mice. Using immunohistochemistry, we found that the phosphorylated NF-κB was dramatically increased in the CCI vehicle and reversed in the IMMA treated mouse sciatic nerve (Figures 5A,B). ELISA assay was performed using fresh lumbar spinal cord tissue from day 14 after CCI to assess the NF-κB DNA binding activity (Figure 5C). Sciatic nerve injury led to a significant increase of NF-κB activation in the spinal cord compared to the sham, and IMMA treatment reduced NF-κB DNA binding with the absorbance at 450 nm of 0.20, 0.54, and 0.43 in sham, vehicle and IMMA treated groups, respectively.

### PGE_2_ Production Was Attenuated by IMMA Treatment

**FIGURE 6:**
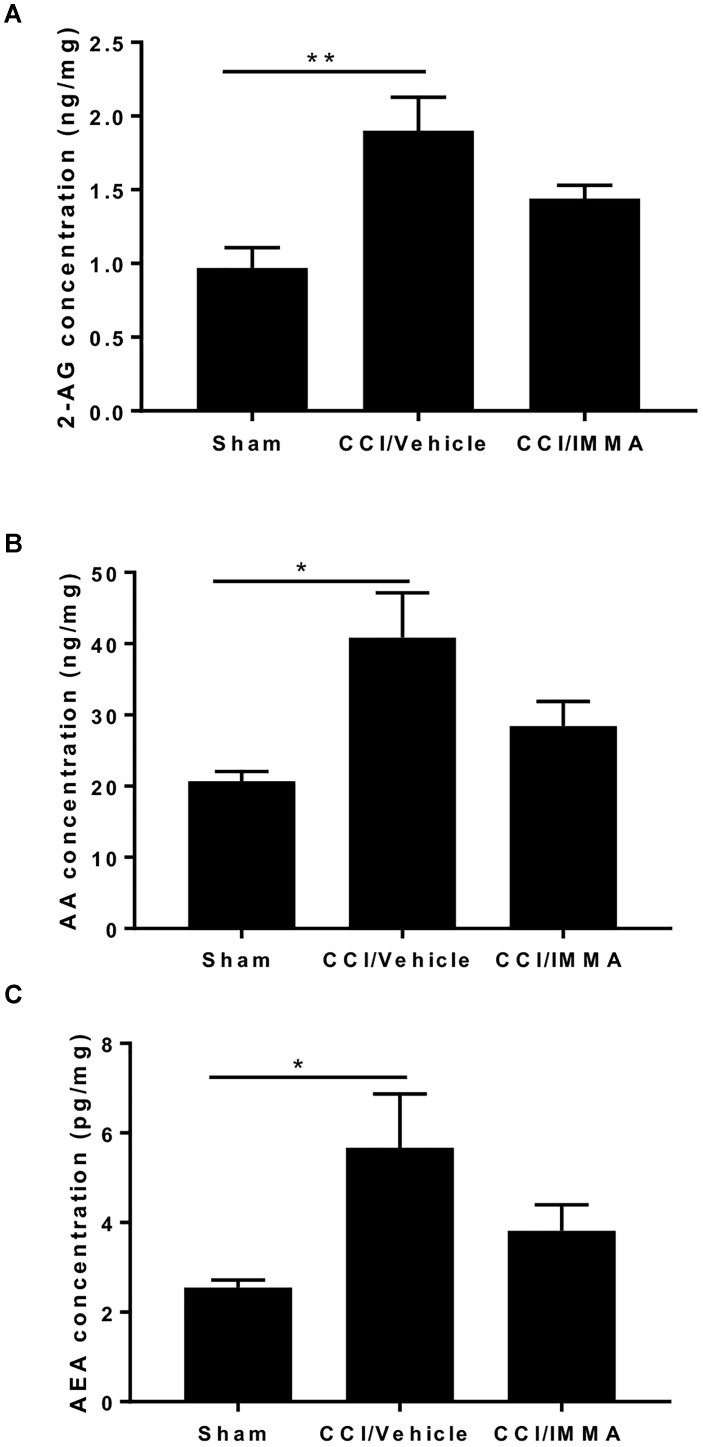
IMMA treatment did not affect the increased levels of 2-AG, AA, and AEA in the CCI mouse sciatic nerve. Fresh sciatic nerve tissues from CCI mice 7 days after surgery were subjected to LS-MS/MS. The amount of 2-AG **(A)**, AA **(B)**, and AEA **(C)** was significantly increased in the CCI/vehicle group compared to the sham (^∗^*p* < 0.05 and ^∗∗^*p* < 0.01. *n* = 8/group). IMMA treatment slightly reduced the increased production of 2-AG, AA, and AEA elicited by CCI **(A–C)**. One-way ANOVA was used for comparing the differences among the sham, vehicle and IMMA treated groups. The Tukey-Kramer *post hoc* test was used for the comparison.

Apart from hydrolytic degradation, endocannabinoids may also be metabolized by cyclooxygenase-2, lipoxygenases, and cytochrome P450, and the metabolites of both AEA and 2-AG also have significant biological functions (Rouzer and Marnett, 2011) To determine whether IMMA, a substrate-selective COX-2 inhibitor can also affect the endocannabinoid metabolism, the levels of 2-AG, AEA and AA in the ipsilateral sciatic nerve after CCI were measured by LC-MS/MS. At 7 days post-injury, there was a significant increase in the levels of 2-AG (1.95 ± 0.23 ng/mg wet tissues), AEA (5.7 ± 1.2 pg/mg wet tissues) and AA (40.83 ± 6.3 ng/mg wet tissues) in the injured sciatic nerve compared to the levels of 2-AG (0.98 ± 0.14 ng/mg), AEA (2.55 ± 0.16 pg/mg) and AA (20.7 ± 1.41 ng/mg) in the sham control animals (^∗^*p* < 0.05, ^∗∗^*p* < 0.01). However, in the IMMA treatment group, no further increase of 2-AG and AEA was observed when compared to the vehicle group, instead, the levels of 2-AG, AEA, and AA were reduced in the IMMA treatment group, although the significant differences were not reached in the vehicle and drug treated groups (Figure 6).

**FIGURE 7:**
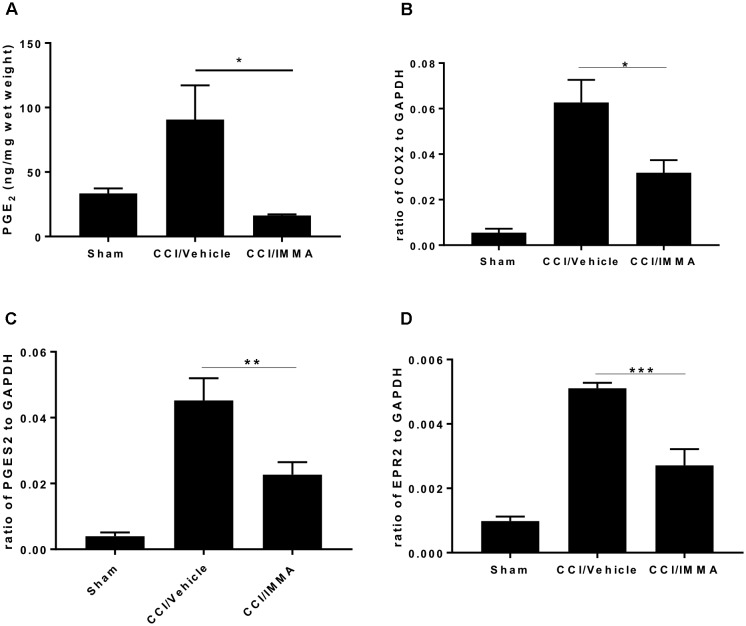
IMMA treatment reduced PGE_2_ synthesis in the CCI mouse ipsilateral sciatic nerve. PGE_2_ production was measured by ELISA with the fresh sciatic nerves from mice 7 days post-CCI. Significantly increased PGE_2_ production in the CCI/vehicle group was remarkably attenuated by IMMA treatment **(A)**. The mRNA expression of enzymes contributing to the PGE_2_ synthesis, COX-2 **(B)** and PGES2 **(C)**, were greatly increased in CCI/vehicle group and reduced by IMMA. The increased expression of EP2 receptor in the CCI/vehicle mice was also attenuated by IMMA treatment **(D)**. ^∗^*p* < 0.05, ^∗∗^*p* < 0.01, and ^∗∗∗^*p* < 0.001 were obtained when the CCI/vehicle group was compared to the IMMA treated group (*n* = 10/group). One-way ANOVA was used for comparing the differences among the sham, vehicle and IMMA treated groups. The Tukey-Kramer *post hoc* test was used for the comparison.

To determine whether treatment with IMMA can affect the AA metabolism, we measured the production of PGE_2_ levels in the ipsilateral sciatic nerve at 14 days post-injury and found a significant increase of PGE_2_ in the CCI/vehicle group (90.7 ± 26.5 ng/mg wet weight) compared to the sham control group (33.51 ± 3.9 ng/mg). Treatment with IMMA reduced PGE_2_ levels to 16.3 ± 1.9 ng/mg, which was significantly lower than the value in the CCI vehicle group (Figure 7A). To elucidate the possible mechanism underlying IMMA mediated PGE_2_ attenuation, we examined mRNA expression of COX-1, COX-2, the PGE_2_ synthases PGES1 and PGES2, and four PGE_2_ receptors, EP1 to EP4 in ipsilateral sciatic nerve. Fresh sciatic nerve fragment from day 7 after CCI surgery was employed for gene expression analysis. Quantitative RT-PCR results showed that CCI mice had a dramatic increase in COX-2 and PGES2 mRNA levels compared to the sham animals (Figures 7B,C), and the increase was attenuated by IMMA treatment. Among the EP receptor family, only EP2 expression was found to be significantly increased in the CCI vehicle group and this increase was dramatically reduced by IMMA treatment (Figure 7D). There were no significant differences in the expression of COX-1 and PGES1 between the sham and CCI vehicle groups (data not shown).

## Discussion

Although blocking AEA and 2-AG hydrolysis has been well-documented to alleviate inflammatory and neuropathic pain, the role of targeting the other metabolic pathways of the endocannabinoids is still in its infancy. In this study, we found that treatment with IMMA (also called LM-4131), a recently developed SSCI (Hermanson et al., 2013, 2014) significantly attenuated hyperalgesia and mechanical allodynia in CCI induced neuropathic pain. The inflammatory response in several regions of pain initiation and transmission pathways including sciatic nerve, DRG and dorsal spinal cord was dramatically reduced in IMMA treated animals. The therapeutic effect is partially reversed by co-administration of CB2 receptor antagonist and is also attributable to the reduced PGE_2_ production.

It has been widely acknowledged that excessive inflammation in both peripheral and central nervous systems contributes to the initiation and maintenance of persistent pain (Kanngiesser et al., 2012; Grace et al., 2014). Given the well-demonstrated anti-inflammatory effects of endocannabinoids and the SSCI can specifically elevate the endogenous levels of AEA and 2-AG, we anticipated that the improvement of IMMA on neuropathic pain behaviors is due to its suppression to inflammatory response. Indeed, IMMA treated mice displayed greatly reduced macrophage infiltration, decreased mRNA expression of proinflammatory cytokines IL-1β, TNF-α and the production of MCP-1 and IL-6 in the injured sciatic nerve. Accumulated microglia and astrocytes in the spinal cord dorsal horn and DRG were also attenuated with IMMA application. Increased expression of NF-κB and its DNA binding activity in the CCI mouse sciatic nerve and spinal cord dorsal horn was also significantly reduced by IMMA treatment. Consistent with the results obtained from the assessment of neuropathic pain behaviors, the inhibitory effects of IMMA on microglia/macrophage accumulation were also reversed by CB2 receptor antagonists, suggesting that the therapeutic action of IMMA is attributable to its activation of CB2 cannabinoid receptors. This result is also consistent with the previous reports showing that CB2 receptor activation ameliorates, whereas deletion of CB2 receptor exaggerates neuropathic pain (Racz et al., 2008a,b; Brownjohn and Ashton, 2012).

We have previously shown that the endogenous levels of 2-AG and AA are significantly elevated in the injured sciatic nerve (Wen et al., 2018), suggesting that both endocannabinoid and eicosanoid signaling are involved in the pathogenesis of neuropathic pain. This result is also in harmony with the observed synergy between the inhibitors of COX-2 and the AEA hydrolytic enzyme FAAH (Grim et al., 2014; Scarpelli et al., 2016) or the principal 2-AG hydrolytic enzyme MAGL (Crowe et al., 2015). In the present study, we also revealed the increased levels of AEA, 2-AG, and AA in the ipsilateral sciatic nerve at 7 days post-CCI surgery. However, different from our hypothesis, treatment with IMMA did not further elevate the increased levels of AEA and 2-AG. On the other hand, the increased levels of AEA, 2-AG, and AA were slightly decreased in the IMMA treated animals. Although the exact causes are unclear, several possibilities might explain the differences from our current result and the previous findings showing that IMMA selectively elevates the endogenous levels of AEA and 2-AG without affecting the AA metabolism (Hermanson et al., 2013). First, in the study by Hermanson et al. (2013), they found that treatment with IMMA increased AEA levels by 139% and the 2-AG levels by 109% in the ICD mouse brain; whereas in our current study, CCI triggered strong inflammatory response in the sciatic nerve and caused more than twofold increase of 2-AG and AEA compared to that in the sham group. Thus, it is possible that the slightly elevated levels of AEA and 2-AG by IMMA can be masked by the compensative increase of these endocannabinoids in the neuropathic pain conditions. Second, given that DAGLβ is the principal 2-AG synthetic enzyme in the peripheral macrophages (Hsu et al., 2012; Shin et al., 2018), suppression of its expression by IMMA may contribute to the reduced production of 2-AG in the injured sciatic nerve. Third, in addition to be oxygenated by COX-2, AEA and 2-AG can also be oxidized by 15-, 12-, and 5-lipoxygenases (LOX) (Kozak et al., 2002; van der Stelt et al., 2002), and several isoforms of cytochrome P450 (Bornheim et al., 1995; Snider et al., 2009). Therefore, it is possible that the inhibition of COX-2 can shift AEA and 2-AG metabolism from COX to LOX and cytochrome P450 mediated metabolic pathways, which may contribute to the reduced levels of these endocannabinoids.

To determine whether treatment with IMMA can affect the arachidonic acid metabolism, we measured PGE_2_ production and the expression of its synthetic enzymes COX-1/COX-2 and PGES1/PGES2 in the injured sciatic nerve and found that both PGE_2_ production and the mRNA expression of COX-2 and PGES2 were reduced by IMMA treatment. Although we speculated that the CB2 receptor mediated endocannabinoid signaling might contribute to the reduced PGE_2_ production, the conversion of IMMA to its parent molecule indomethacin *in vivo* can not be excluded and therefore it may have a direct inhibitory effect on the COX-2 activity. It has been reported that R-profens can undergo unidirectional inversion to the S-enantiomers *in vivo* (Windsor et al., 2012). The very recent study by Morgan et al revealed that about 10% of LM-4131 can be converted to indomethacin and both R-flurbiprofen and LM-4131 prevent the formation of most PGs and PG-Gs to a similar extent (Morgan et al., 2018). Although R-flurbiprofen has been shown to reduce neuropathic pain by restoring the levels of endocannabinoids in DRG and spinal cord (Bishay et al., 2010), we found that treatment with either S-flurbiprofen or R-flurbiprofen at 10 mg/kg (when used at the same dose as that of IMMA) lead to gastrointestinal bleeding and resulted in mortality in mice, despite the former was more severe (data not shown). The toxicity of R-flurbiprofen is likely due to its conversion to S-flurbiprofen *in vivo* (Schmitz et al., 2014). Thus, we believe that IMMA is a safer SSCI compared with the R-enantiomer of flurbiprofen.

Taken together, our results showed that IMMA administration significantly alleviated hyperalgesia and mechanical allodynia in the CCI mouse model by potential novel mechanisms. It is likely that enhancement of endocannabinoid signaling and reduction of inflammatory PGE_2_ production may both contribute to the therapeutic effects of IMMA in neuropathic pain. Therefore, we believe that IMMA is a promising therapeutic agent for pain relief, despite that the underlying mechanisms that contribute to the therapeutic efficacy of IMMA and other SSCIs remain to be elucidated.

## Author Contributions

MJ, PS, and MT performed the experiments and analyzed the data. SM assisted the endocannabinoid and arachidonic acid measurement using mass spectrometry and the data analysis. MJ and JW prepared the figures. JW and YZ wrote the manuscript. YZ designed the experiments and secured funding for the studies. All authors read and approved the final manuscript.

## Conflict of Interest Statement

The authors declare that the research was conducted in the absence of any commercial or financial relationships that could be construed as a potential conflict of interest.
